# Proteomics of HRpQCT Bone Loss in Older Men

**DOI:** 10.1093/geroni/igaf122.472

**Published:** 2025-12-31

**Authors:** Eric Orwoll, Adam Burns, Jack Wiendrick, Alicia Feryn, Andrew Burghardt, Jodi Lapidus

**Affiliations:** Oregon Health & Science University, Portland, Oregon, United States; Oregon Health & Science University, Portland, Oregon, United States; Oregon Health & Science University, Portland, Oregon, United States; Oregon Health & Science University, Portland, Oregon, United States; University of California, San Francisco, San Francisco, California, United States; Oregon Health & Science University, Portland, Oregon, United States

## Abstract

The biological basis of bone loss in older people is unclear, and current biomarkers are of limited usefulness. We measured serum protein levels (SomaLogic 7K panel) in 230 men (84 + 3 years) and determined associations with HRpQCT change (distal radius, distal tibia) over ∼6 yrs, with adjustments for time between measures, age, height, weight and genomic principal components. Primary skeletal outcomes were total vBMD and failure load. Linear regression of proportional change in HRpQCT was used to assess protein associations. 16 proteins were robustly associated with skeletal change, 7 at the radius and 10 at the tibia, with only one protein associated with change at both measurement sites. In almost all cases, higher proteins levels were related to increased bone loss. Associations at the radius and tibia were concordant in direction, and associations with change in total vBMD and failure load were essentially the same. Associations with changes in DXA hip BMD were similar. FSH levels were most strongly (negatively) related to change. A 19-protein signature was much more strongly predictive of bone loss than traditional bone remodeling markers (C statistics: signature - 0.83, CTX – 0.58, P1NP – 0.54). In sum, serum protein levels were strongly associated with skeletal change, the association patterns were distinct at the radius and tibia, suggesting the biological pathways governing bone change at the two sites are unique. A protein signature may represent a powerful biomarker of bone loss.

